# Exploring the effectiveness of proactive service models in preventing medical disputes and petitions: a pilot investigation using a before-and-after design at a single center in Hangzhou, China

**DOI:** 10.3389/fpubh.2026.1765109

**Published:** 2026-03-09

**Authors:** Yihang Yang, Zhezhong Zhang, Shiliang Chen, Xiaohong Liu, Lanzhi Zheng, Shuwei Huang, Yang Lu

**Affiliations:** The First Affiliated Hospital of Zhejiang Chinese Medical University (Zhejiang Provincial Hospital of Chinese Medicine), Hangzhou, Zhejiang, China

**Keywords:** communication resolution rate, incidence of petitions, medical disputes, medical petitions, proactive service model

## Abstract

**Background:**

Healthcare conflicts are a global public health priority, and medical petitions are a primary resolution pathway in China. Zhejiang Provincial Hospital of Chinese Medicine (ZPHCM) found that a reactive administrative approach failed to curb clinical disagreements and petition frequency, requiring optimized management strategies.

**Methods:**

A single-institution before-and-after observational pilot study (no concurrent control) was conducted across ZPHCM’s Qiantang and Hubin campuses. The Proactive Service Model (PSM) was initiated in January 2025; outcome metrics were compared between an 8-month pre-intervention period and a 9-month post-intervention period. Analyses were performed using IBM SPSS Statistics 27.0 and R v.4.4.3 (descriptive statistics, Cohen’s d, Mann–Whitney U tests).

**Results:**

Post-PSM deployment, both campuses showed significant improvements: Qiantang Campus had communication resolution rate (CRR) increased from 35.95% ± 13.02 to 52.97% ± 8.22 (+17.02 percentage points) and petition incidence rate (IP) decreased from 34.88% ± 6.89 to 27.67% ± 5.47 (−7.20 points); Hubin Campus had CRR raised from 46.02% ± 8.55 to 57.87% ± 5.75 (+11.85 points) and IP reduced from 33.48% ± 7.73 to 25.85% ± 5.29 (−7.63 points). Effect sizes were substantial (CRR: Qiantang d = 1.59, Hubin d = 1.65; IP: both d = −1.17) with 95% CIs excluding zero, and CRR changes were statistically significant (U = 9, *p* = 0.011).

**Conclusion:**

As a pilot study, this work lacks definitive causal evidence, but it demonstrates a meaningful association between PSM implementation and improved communicative dispute resolution alongside reduced petition incidence at ZPHCM, supported by notable effect sizes.

## 1 Introduction

Globally, medical conflicts have emerged as a significant public health concern in recent years, attracting substantial scholarly and policy attention (1). The term “medical petitions” denotes a procedure wherein individuals circumvent internal grievance mechanisms at healthcare facilities to lodge complaints directly with governmental authorities. Within the Chinese context, this petition system operates as an integral component of the healthcare dispute resolution framework, furnishing avenues for public expression and institutional monitoring (2, 3). Although this mechanism effectively addresses societal grievances and propels enhancements in the healthcare sector, it concurrently tarnishes the reputation of hospitals and amplifies their operational expenditures and strains (4, 5).

In China, friction between healthcare providers and patients is intensifying. Empirical data indicate that roughly 62% of medical professionals and patients encounter disputes in some form (6, 7). Key contributory elements include: first, the broadening of fundamental health insurance coverage, which has expanded service accessibility (8, 9)—with enrollment rates escalating from 11.97% in 2006 to 60% in 2012 and surpassing 95% by 2021—while simultaneously heightening public anticipations for care quality and interpersonal dynamics in clinical settings (10, 11); secondly, imbalances in medical expertise between clinicians and patients, compounded by trust deficiencies, which foster resistance to investigative processes and mediation offers from dispute resolution bodies, thereby aggravating tensions (12, 13); and third, deficient communication practices, insufficient competencies in dialogue, and a marginalized recognition of interaction importance among care providers and patients or their relatives (14, 15). Medical disputes represent a ubiquitous challenge affecting health systems worldwide, with profound repercussions for care standards, the welfare of healthcare personnel, patient confidence, and the operational efficacy and endurance of healthcare infrastructures (16, 17).

In China, the healthcare system utilizes a structured classification model comprising three tiers and ten grades (18, 19). Under this scheme, tertiary medical institutions are differentiated into Class A, Class B, and Class C based on their functional capabilities, with Class A representing the top echelon (4, 20). As an institution that has attained Class A tertiary designation through formal evaluation, ZPHCM resides at the pinnacle of this medical service structure. Hence, the prevention and management of practitioner-patient conflicts have become a critical priority for hospitals in evolving their clinical systems.

Globally, the management of healthcare conflicts manifests in three dominant frameworks: firstly, disclosure-and-resolution programs that prioritize openness in communication and timely settlement to avoid court proceedings (21, 22); secondly, mediator-facilitated medical dialogue systems employing specialized neutrals to reconcile differing interests (23); and thirdly, multi-tiered administrative review processes where independent medical boards address conduct-related grievances (24). These approaches, however, are largely reactive, concentrating on post-incident remediation and depending on well-institutionalized Alternative Dispute Resolution (ADR) structures or professional self-governance (25). Within the distinctive context of China’s administrative petitioning apparatus, PSM investigated here incorporates anticipatory communication pathways, mechanisms for shared institutional liability, and formalized quality oversight. This synthesis creates a comprehensive management system focused on preempting conflict escalation, thereby providing a tailored resolution framework for high-capacity public hospitals that diverges from conventional international models.

The present study objectives are: (1) To discern hurdles across distinct phases of medical disputes and craft efficacious remedies; (2) To advance the hospital’s clinical infrastructure, elevate patient contentment, and diminish the frequency of petitions; (3) To examine the contribution of PSM in precluding and addressing medical disputes and related petitions.

## 2 Materials and methods

### 2.1 Data collection

This pilot investigation was implemented across the Qiantang and Hubin campuses of ZPHCM, organized into two sequential stages. During the first phase, the Patient-Physician Communication Center (PPCC) engaged in initial dialogues with patients to ascertain event particulars, classify the nature of the occurrence, and document patient appeals. These exchanges were held in private consultation settings. When mutual understanding was attained and the patient opted against further escalation, the situation was categorized as a Communication Resolution Case (CRC). In cases where accord was not achieved, and the individual formally submitted their concern via the hospital’s established internal grievance system, it was designated as a Complaint. Should the patient advance their case through governmental agencies as per legal frameworks—whether or not an internal complaint was also submitted—it was registered as a Petition.

In the subsequent conflict resolution stage, for events where the healthcare institution and patient remained at an impasse, the Medical Affair Department (MAD) cooperates with the implicated clinical department to assemble a dedicated task force (TF) for exhaustive monitoring. TF is mandated to first concentrate on recording three fundamental components: the primary disputed elements of the event and the patient’s exact requirements; the methodology employed for problem resolution and the conclusive action plan; and the regulatory justification for plan execution. At the same time, vital original materials, including medical records, interaction logs between doctors and patients, and settlement agreements, require preservation. By reinforcing holistic oversight of the evidence pathway, a closed and auditable evidentiary circuit is formed.

These monitoring documents are required to rigorously comply with the 5W1H principle (What, Who, When, Where, Why, How) to determine the originating source of the event. After the dispute is conclusively settled, distinct monitoring intervals are instituted depending on the event category. Priority is placed on obtaining patient evaluations of the resolution effectiveness, while simultaneously compiling any supplementary appeals or proposals for refinement to direct later managerial enhancements.

To incorporate the dimension of patient-reported outcomes, aggregated metrics were derived from routine satisfaction surveys administered by PPCC. Data from the full year 2024 (pre-intervention) were compared with results from the three subsequent quarters of 2025 (post-intervention). These surveys, executed independently for outpatient and inpatient services, employed consistent instrumentation and identical sampling protocols. Our analysis focused on the quarterly aggregate proportion for “Overall Satisfaction,” defined as the combined percentage of responses categorized as ‘Satisfied’ and ‘Very Satisfied.’ Descriptive comparisons are presented stratified by department. Statistical evaluation of pre- versus post-intervention quarterly data was performed using paired t-tests.

### 2.2 Data analysis

All interactions with patients at the PPCC are to be recorded only after securing explicit consent, thereby fully protecting their right to make informed decisions. These audio records require immediate transcription into textual format for archiving. This method of data gathering is inherently non-reproducible, as it is contingent upon unique communicative contexts and individualized patient permissions. The Management Weekly Report (MWR) is tasked with compiling a weekly summary of the accumulated data, which includes Event Types, Quantities, Underlying Causes, and implemented Resolutions.

For analytical purposes, CRR is calculated as the proportion of monthly CRC cases relative to the entire volume of dispute incidents, which encompasses CRC, Complaints, and Petitions. Conversely, IP is defined as the share of Petitions out of the total dispute incidents. This investigation adopted a single-site, before-and-after design, with the initiation of PSM in January 2025 serving as the demarcation point. Outcome metrics from an 8-month pre-intervention phase were compared with those from a 9-month post-intervention phase. All analyses were conducted using IBM SPSS Statistics 27.0 and R software (version 4.4.3), with a two-tailed alpha level of *α* = 0.05 defined for significance. To account for potential serial correlation in the monthly measurements, a dual analytical approach was implemented to appraise the intervention’s impact.

#### 2.2.1 Descriptive and effect size analysis

Means and standard deviations were computed for both CRR and IP, along with absolute change values. The standardized mean difference, quantified by Cohen’s d (d = (M_post − M_pre)/SD_pooled), served as the principal metric for interpreting the intervention’s magnitude. Corresponding 95% confidence intervals were generated. In line with established benchmarks, absolute values of d below 0.2 were considered negligible, from 0.2 to 0.5 small, 0.5 to 0.8 moderate, and exceeding 0.8 large. This measure was prioritized for its clinical interpretability and relative independence from sample size.

#### 2.2.2 Statistical significance testing

The nonparametric Mann–Whitney U test was applied to evaluate shifts in central tendency. This method was selected for its robustness to distributional assumptions and potential time-series dependencies, making it appropriate for the limited sample size and repeated measurement context.

#### 2.2.3 Autocorrelation assessment

Time dependency within the data series was examined using the Durbin-Watson statistic. The limited sequential data points (*n* = 8–9 per period) precluded formal statistical adjustment; this constraint is explicitly acknowledged as a study limitation.

Furthermore, to incorporate a supplementary qualitative dimension, core narrative themes were identified from incident records within regular MWR. Illustrative cases were selected for presentation based on two primary criteria: (1) clear exemplification of defined intervention mechanisms, and (2) documentation of a complete, representative event trajectory. It is critical to note that only cases culminating in successful resolution were chosen for mechanistic illustration. Consequently, this purposive selection does not support efficacy claims and is intended solely for descriptive and exploratory purposes.

### 2.3 Figure preparation

**Figure 1:**
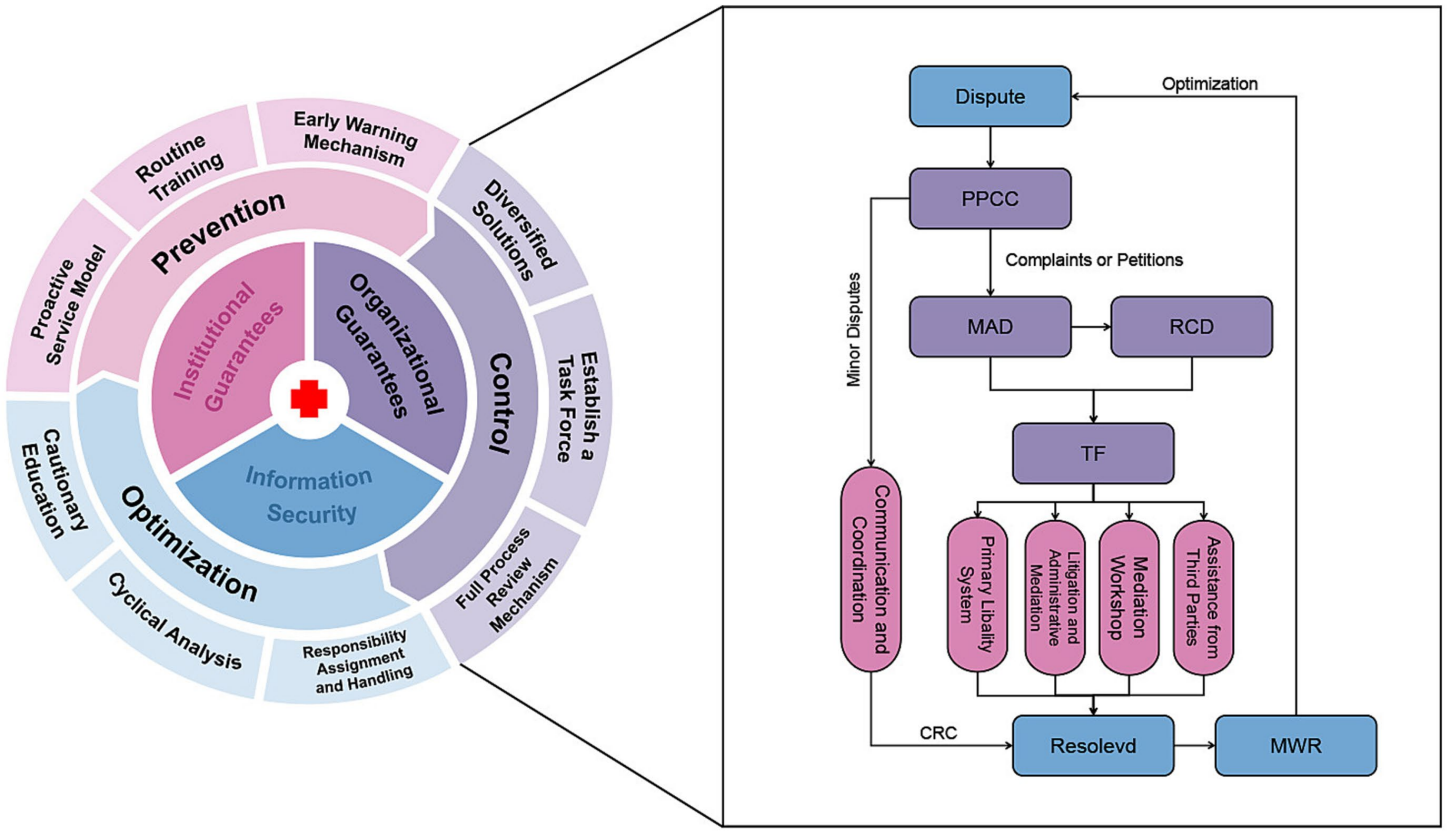
Delineates the systematic workflow and key interventions of ZPHCM’s integrated framework for the prevention, handling, and continuous improvement of medical disputes. Upon the occurrence of an incident, the PPCC initiates immediate mediation, aiming to resolve straightforward issues at the point of care. For cases where patients pursue their grievances via the hospital’s internal complaint procedure or through a formal petition to governmental bodies, the MAD assumes responsibility. A specialized TF, which includes representatives from the involved clinical department and the RCD, is then activated to deploy coordinated, multi-dimensional strategies. The process concludes with the aggregation of root cause analyses and final outcomes into the MWR for reporting and review. PPCC, Patient-Physician Communication Center; MAD, Medical Affair Department; RCD, Relevant Clinical Department; TF, Task Force; MWR, Management Weekly Report; CRC, Communication Resolution Case.

Figures in this study were produced using different software tools as follows:

Developed using Microsoft PowerPoint (Microsoft Corporation, Redmond, WA, USA). The application facilitated the construction of the diagram’s architecture, where components including its organization, structural relationships, and procedural flow were configured to optimize clarity and support effective visual communication (see Figure 1).

**Figure 2:**
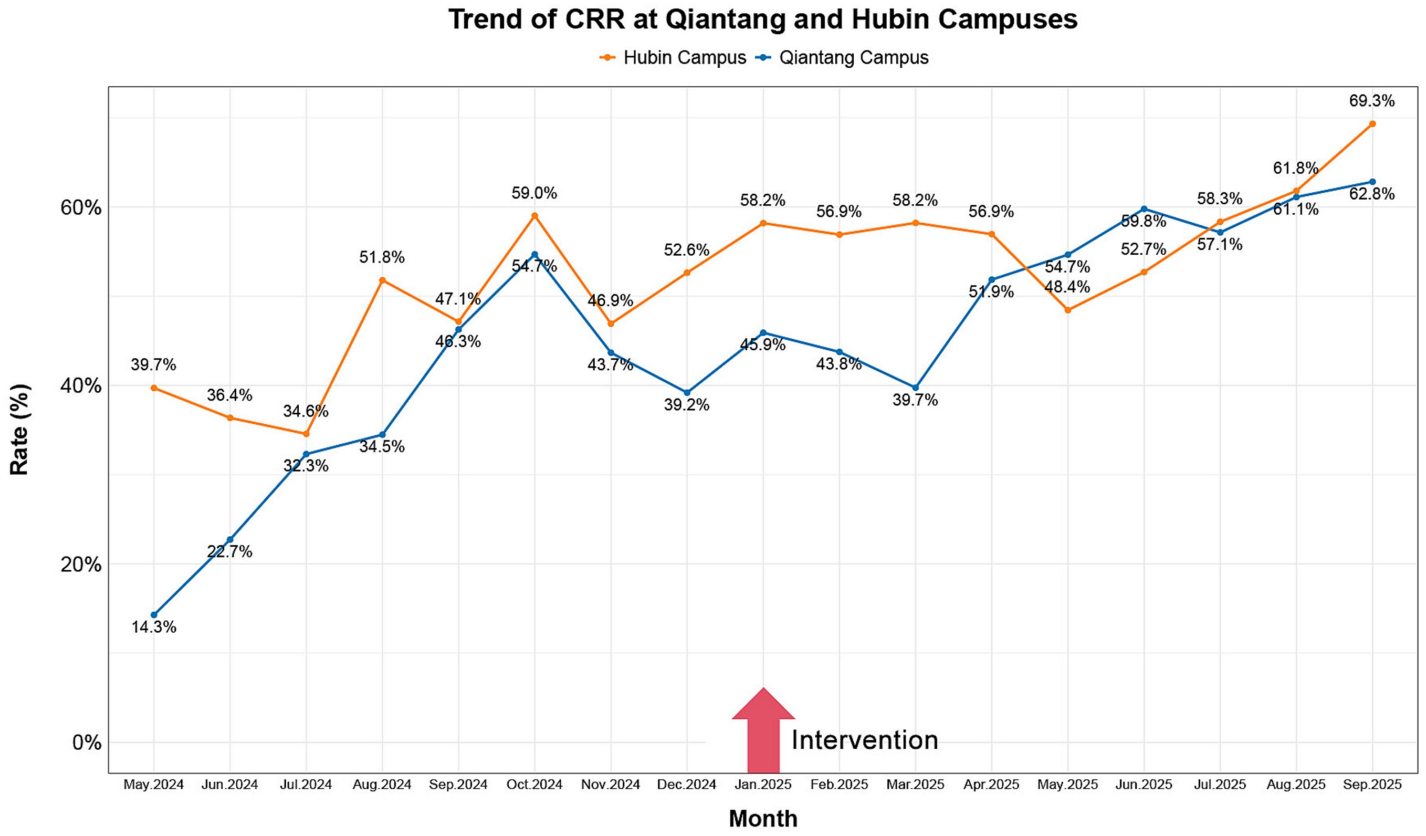
A 17-month trend analysis of campus-wide CRR pre- to post-intervention. In the presented figure, the yellow line indicates the trend of CRR at Hubin Campus, while the blue line indicates the trend of CRR at Qiantang Campus. CRR, communication resolution rate.

**Figure 3:**
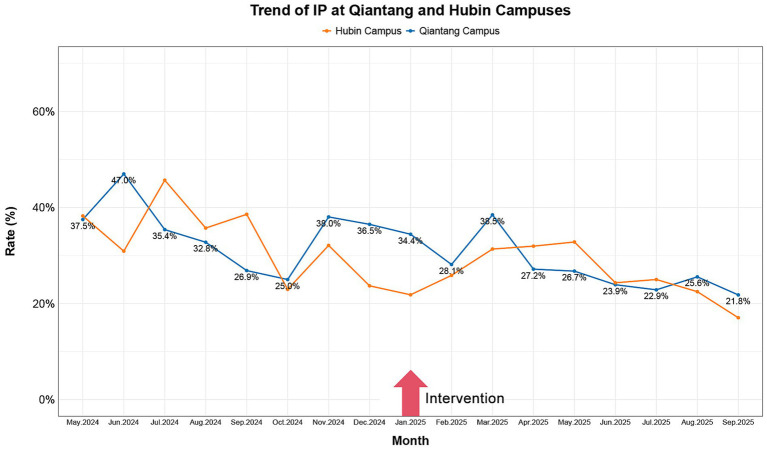
A 17-month trend analysis of campus-wide IP pre- to post-intervention. In the presented figure, the yellow line indicates the trend of IP at Hubin campus, while the blue line indicates the trend of IP at Qiantang campus. IP, incidence of petitions.

**Figure 4:**
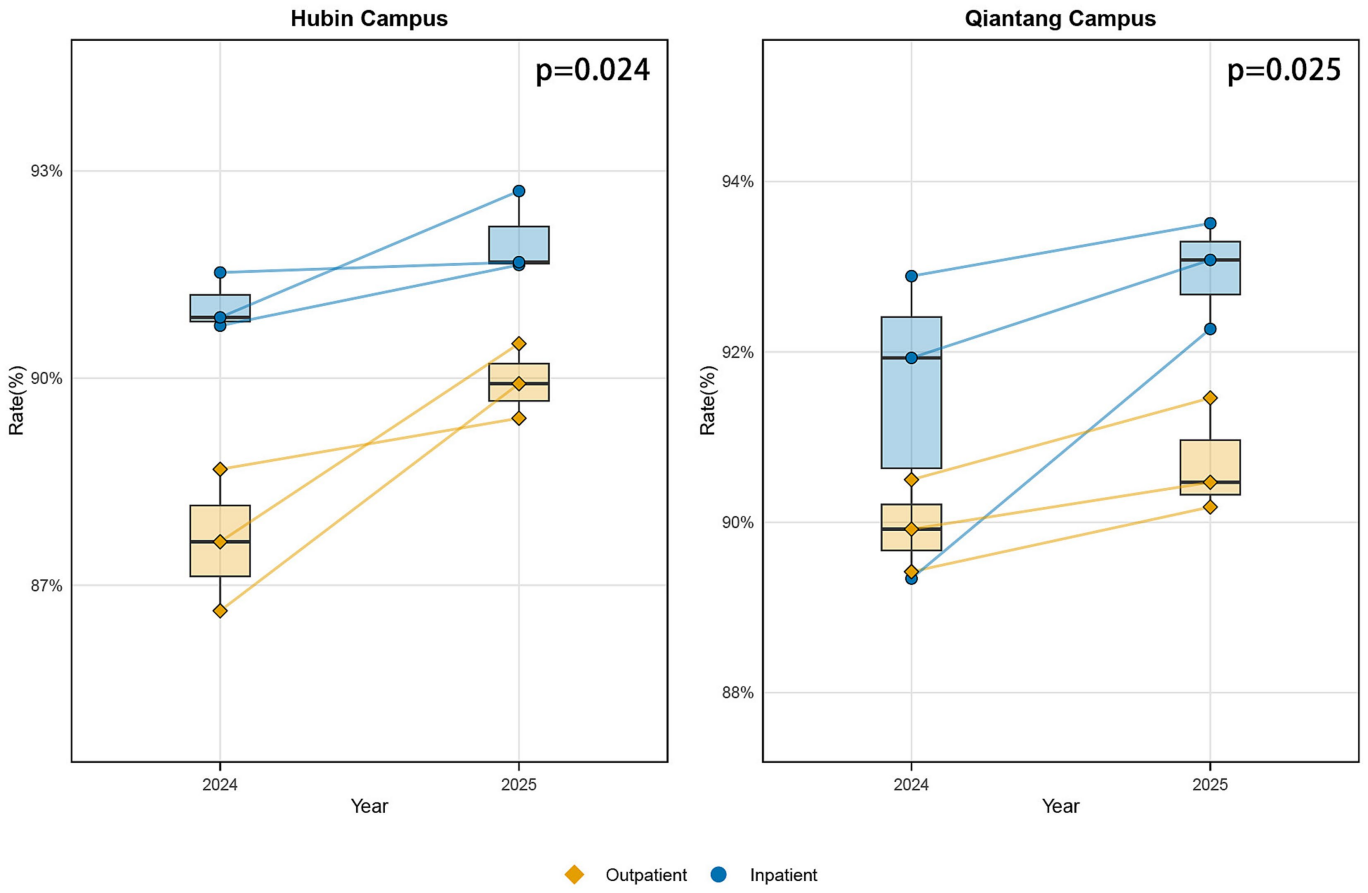
Paired boxplots comparing patient satisfaction changes between Qiantang and Hubin campuses (paired analysis). The left graph shows Hubin campus, while the right graph shows Qiantang campus. Data for each campus is stratified by outpatient (diamond markers) and inpatient (circle markers) departments, illustrating changes in the overall satisfaction rate between 2024 (pre-intervention) and 2025 (post-intervention). The lines connect paired quarterly data, illustrating intra-departmental temporal trends. Paired comparisons (*n* = 3 pairs) across three quarters for each outpatient and inpatient department at each campus used a paired *t*-test to assess satisfaction changes. Outpatient, Outpatient Department; Inpatient, Inpatient Department.

These visualizations were constructed utilizing R software, version 4.4.3 (R Foundation for Statistical Computing, Vienna, Austria). Analytical workflows employed specialized R packages for data processing and graphical generation. All visual components, such as trend lines, annotations, and legends, were meticulously defined within the software environment to guarantee both precise representation and enhanced interpretability of the plotted data (see Figures 2–4).

All figures were exported in a high-resolution format (JPEG, 1000 dpi) to maintain clarity for subsequent manuscript processing and review.

Key outcome metrics before and after implementation of the PSM. Data were processed and analyzed using R software, version 4.4.3 (Table 1).

**Table 1:** Comparison of outcome measures before and after intervention.

Variable	Campus	Pre-intervention (*M* ± SD)	Post-intervention (*M* ± SD)	Difference	Cohen’s d (95% CI)	Mann–Whitney U	D-W
CRR	Qiantang	35.95 ± 13.02	52.97 ± 8.22	17.02	1.59 (0.48, 2.69)	9, *p* = 0.011	0.493
	Hubin	46.02 ± 8.55	57.87 ± 5.75	11.85	1.65 (0.53, 2.77)	9, *p* = 0.011	1.144
IP	Qiantang	34.88 ± 6.89	27.67 ± 5.47	−7.2	−1.17 (−2.21, −0.13)	56, *p* = 0.061	1.351
	Hubin	33.48 ± 7.73	25.85 ± 5.29	−7.63	−1.17 (−2.21, −0.13)	56, *p* = 0.061	1.507

Cohen’s d effect size calculated based on pooled standard deviation; 95% CI = 95% confidence interval. Mann–Whitney U test is robust to time dependence; D-W = Durbin-Watson statistic (2 = no autocorrelation, <2 positive autocorrelation, >2 negative autocorrelation). *p < 0.05 indicates statistical significance, †*p* < 0.10 indicates marginal significance. (i.e., not significant at the strict 0.05 significance level but still interpretable) CRR, Communication Resolution Rate; IP, Incidence of Petitions.

## 3 Result

### 3.1 Practical case studies of PSM

This case study is based on typical sampling and aims to illustrate the operational mechanisms of PSM. It does not represent the outcomes of all disputes. Since MWR does not systematically record unresolved cases, it is impossible to assess differences between typical cases and the overall population.

To actively identify and resolve patient concerns prior to their escalation into formal disputes, ZPHCM has implemented the telephone complaint system (TCS), a multichannel framework incorporating a WeChat mini-program, online portal, telephone, email, and SMS. An empirical case example is provided below.

A patient, Mr. Wang, presented at the hospital’s emergency dog bite clinic. Anxious that a prolonged wait would compromise his treatment, he approached a physician for clarification. He was informed that a single doctor was concurrently covering both the emergency surgery department and the dog bite clinic, resulting in slower service. Mr. Wang voiced his dissatisfaction and considered lodging a grievance via the Mayor’s hotline. However, he noticed a dedicated complaint hotline posted outside the clinic and decided to call that number instead. Personnel from the Patient-Physician Communication Office promptly responded to manage the situation. In the end, Mr. Wang was content with the institution’s responsive service and resolution, leading him to forego any further formal petition.

This incident demonstrates how the TCS at ZPHCM successfully averted a potential medical conflict. The system facilitated internal mediation that directly addressed the patient’s clinical concerns and resolved the underlying issue.

Beyond concerns related to outpatient services, surgical complications after procedures often become sources of formal grievances from patients and their families. To ensure patient safety during the entire course of therapy and recovery after surgery, ZPHCM has adopted the surgical liability insurance (SLI) initiative. An illustrative case is described herein.

A patient, identified as Han, completed all necessary preoperative evaluations and was admitted for a laparoscopic left hepatectomy combined with abdominal lymph node dissection. Following the surgery, the patient was moved to a standard ward. During postoperative monitoring, a significant hypotension episode occurred, with blood pressure readings falling to 70–60/30–40 mmHg. A point-of-care ultrasound examination raised suspicion of an abdominal hemorrhage. Subsequent to a multidisciplinary review and discussions with the family members, a second exploratory surgery was performed to secure hemostasis. This follow-up operation managed to successfully arrest the bleeding. The family later voiced allegations concerning bleeding during the initial surgery and lodged a formal complaint with the Medical-Patient Relations Office. Because the patient had previously enrolled in the SLI plan before the operation, the affiliated insurance provider stepped in. The ensuing claim was adjudicated based on the stipulations of the pre-existing insurance policy. Ultimately, the patient reported a positive assessment of the SLI claims administration and support, which resulted in the formal retraction of the complaint.

This incident demonstrates that the SLI framework instituted by ZPHCM allows for prompt insurance participation in both clinical issue resolution and financial claim settlement, which helps prevent the escalation of disputes and promotes higher levels of patient satisfaction.

In the phase subsequent to dispute resolution, ZPHCM protocol necessitates that MAD work in conjunction with the Relevant Clinical Department (RCD) to assemble a TF for supervising the conflict’s evolution. This methodology serves to meet patient expectations while curbing the advancement of formal grievances. An illustrative instance is provided below.

A patient, Mr. Zheng, who had undergone pituitary tumor resection 22 days earlier at an external facility, scheduled a follow-up cortisol assessment at the neurosurgery outpatient clinic. The procedure was intended for approximately 8:00 a.m., with the patient arriving at 7:55 a.m. By 10:00 a.m., the individual exhibited considerable agitation and enumerated multiple concerns: the attending physician did not reach the clinic until 8:33 a.m.; the clinician displayed an unsatisfactory bedside manner by instructing the patient to seek subsequent care at the original hospital; when asked about the timeliness of the test, the physician referred the query to the laboratory staff; and upon soliciting an interpretation of required tests, the doctor professed an inability to analyze the results and advised comparison with prior records from the other institution. Owing to extreme frustration, the patient was relocated to the emergency department. The individual then proceeded to the PPCC, insisting on stringent sanctions against the physician and threatening to utilize the mayor’s hotline.

A review substantiated that the patient’s narrative was predominantly accurate. The MAD subsequently extended apologies and reassurances, facilitating a consultation with a neurosurgery inpatient specialist. This specialist diligently engaged with the patient’s issues and prescribed the necessary follow-up evaluations. As a result, the patient indicated satisfaction and rescinded the planned petition.

This episode demonstrates that the synergistic efforts of MAD and PPCC successfully forestalled the initiation of a formal complaint. Furthermore, the fundamental origins of the disagreement highlighted persistent institutional weaknesses within ZPHCM. By adopting refined operational frameworks, the organization is committed to steadily strengthening the foundational architecture of its clinical care system (Figure 1).

### 3.2 Analysis of the effectiveness of introducing PSM to improve CRR

Subsequent to the adoption of the PSM, CRR at both hospital campuses demonstrated a notable increasing trajectory, as visually detailed in Figure 2 and numerically in Table 1. Specifically, the Qiantang Campus exhibited a rise in CRR from a pre-intervention level of 35.95% (±13.02) to 52.97% (±8.22) post-intervention, constituting an absolute increase of 17.02 percentage points. Concurrently, the Hubin Campus saw its CRR elevate from 46.02% (±8.55) to 57.87% (±5.75), an 11.85 percentage-point gain. Analysis of effect sizes revealed large-magnitude changes for both locations (Qiantang: Cohen’s d = 1.59, 95% CI: 0.48–2.69; Hubin: d = 1.65, 95% CI: 0.53–2.77). The fact that neither confidence interval included zero suggests these are clinically meaningful changes. Statistical significance for these shifts was further corroborated by Mann–Whitney U test results (U = 9, *p* = 0.011).

### 3.3 Analysis of the effectiveness of introducing PSM to reduce IP

The observed trend in petition incidence corresponds with anticipated outcomes, albeit demonstrating less robust statistical significance when compared to the CRR results (refer to Figure 3 and Table 1). At Qiantang Campus, IP declined from 34.88% (±6.89) to 27.67% (±5.47), reflecting an absolute reduction of 7.20 percentage points. A similar decrease was observed at Hubin Campus, where the IP fell from 33.48% (±7.73) to 25.85% (±5.29), a change of −7.63 percentage points. Analysis indicated a large effect size for this reduction at both sites (d = −1.17, 95% CI: −2.21 to −0.13). However, the proximity of the confidence interval’s lower bound to zero points to considerable estimation uncertainty. Statistical testing via the Mann–Whitney U test yielded *p*-values of 0.061 for each campus, a result marginally above the conventional significance threshold. This borderline non-significance likely reflects constraints in statistical power attributable to the limited sample size in this pilot investigation.

### 3.4 Changes in patient satisfaction

Subsequent to the introduction of PSM, metrics for patient satisfaction pertaining to both outpatient and inpatient services at the Qiantang and Hubin campuses exhibited a consistent pattern of improvement. All comparable quarterly measurements showed directional alignment, with no periods of decline observed (Figure 4). More specifically, aggregate satisfaction at the Qiantang campus rose from 90.67% (±1.44%) during the 2024 baseline period to 91.83% (±1.36%) in the 2025 follow-up interval, marking an increase of 1.16 percentage points. Correspondingly, the Hubin campus recorded an elevation from 89.35% (±1.99%) to 90.98% (±1.24%), equating to a gain of 1.63 percentage points. Analyses using paired t-tests confirmed that these shifts were statistically significant (Qiantang: *t* = 3.17, df = 5, *p* = 0.025, d = 1.29; Hubin: *t* = 3.18, df = 5, *p* = 0.024, d = 1.30). The calculated Cohen’s d values for both campuses indicate a large magnitude of effect. It should be noted that formal statistical comparisons of the satisfaction change magnitudes between the two clinical departments were not performed within the scope of this analysis.

## 4 Discussion

This pilot investigation utilized a single-center, before-and-after observational design, demarcated by the implementation of PSM in January 2025. It systematically compared key outcome metrics across an 8-month pre-intervention and a 9-month post-intervention period. Statistical techniques were chosen in accordance with the specific attributes of the data and the exploratory aims of the research. By contextualizing the study within established international paradigms for managing healthcare conflicts, it clarifies the positioning, potential integration avenues, and inherent constraints of the PSM, thereby offering a methodological and practical reference for subsequent inquiry and application.

The selection of this research design was guided by considerations of its exploratory purpose, ethical parameters, and data feasibility. As a pilot study, its primary objectives were to evaluate implementation practicality, gauge preliminary effect trends, and generate hypotheses—not to definitively establish causality (26). The pre-post design, coupled with effect size analysis, offers procedural simplicity and effectively captures directional changes, aligning with these exploratory aims. Given that PSM was instituted as a hospital-wide quality improvement initiative involving systemic procedural and staffing adjustments, randomized assignment or parallel control groups were not feasible (27). The chosen design minimizes baseline comparability issues while adhering to ethical and practical constraints. The research leveraged the institution’s established MWR, which furnishes a continuous, standardized source of monthly dispute-related data characterized by temporal continuity, representativeness, and complementary outcome measures. Core monthly metrics—CRR and IP—were supplemented by quarterly patient satisfaction data to appraise intervention effects from dual perspectives: dispute management efficiency and patient experience.

The principle guiding statistical method selection was their congruence with data properties and research questions. Acknowledging the time-series nature of the monthly data, limited sample size, and pilot objectives, the analysis prioritized the Mann–Whitney U test and effect size estimation (Cohen’s d), foregoing robust autocorrelation corrections like Newey-West. This decision was rigorously justified: Durbin-Watson statistics indicated autocorrelation, and the small sample size (*n* = 8–9 per period per campus) violated assumptions for parametric tests. The nonparametric Mann–Whitney U test, robust to distributional assumptions and less sensitive to autocorrelation, was deemed appropriate for small samples (28). Furthermore, for a pilot study, the clinical magnitude of an effect (effect size) is often more informative than statistical significance alone. Cohen’s d provides a sample-size-independent measure of this magnitude (29). The observed large effect sizes for CRR increase and IP reduction meaningfully reflect PSM’s preliminary impact.

Interrupted Time Series (ITS) or piecewise regression were not employed due to insufficient pre-intervention data points (fewer than the recommended 12–15) (30, 31), which would lead to model instability and biased estimates. The heterogeneous autocorrelation patterns between campuses further complicated such modeling. The simpler, robust analytical strategy adopted was consistent with the study’s exploratory scope.

The reporting adheres to STROBE guidelines, employing a dual interpretive strategy that emphasizes clinical relevance (via effect size) while transparently disclosing statistical uncertainty. Post-intervention, CRR demonstrated large, statistically significant improvements (Qiantang d = 1.59; Hubin d = 1.65; *p* = 0.011). IP also showed large-magnitude reductions (d = −1.17 for both), though the associated *p*-values (0.061) marginally exceeded the significance threshold, a result interpreted in light of sample size limitations.

The study acknowledges several constraints: the lack of a parallel control group limits the ability to attribute changes solely to PSM, as historical or concurrent factors cannot be ruled out (32, 33). The complex temporal data structure and autocorrelation also necessitate cautious interpretation. The observed strong negative autocorrelation in Qiantang’s CRR, potentially indicative of a mean reversion effect, warrants further investigation.

Based on these insights, four directions for future research are proposed: (1) extending the baseline observation period and adopting a formal ITS design in subsequent studies; (2) implementing a multicenter or stepped-wedge randomized design to strengthen causal inference (34); (3) enriching outcome measures with patient-reported outcomes (PROs); and (4) integrating qualitative methods to explore implementation mechanisms and contextual factors (35).

When benchmarked internationally, PSM—focusing on proactive prevention integrated with administrative systems—presents a model adapted to China’s institutional context, where administrative petitions are a predominant dispute channel. It complements established paradigms like the U.S. Disclosure and Resolution Program (DRP) by providing a front-end communication trigger (21, 22), aligns with Japan’s Medical Communication Officer system through specialized personnel training (23), and could function as a filtering layer within Singapore’s tiered regulatory framework (24). Its sustainability and broader integration require further institutional support and long-term data.

In conclusion, this pilot study indicates the feasibility and promising preliminary effects of PSM in enhancing communication resolution and potentially reducing formal petitions. By transparently justifying its methodological choices and delineating its limitations, the research provides a foundational model and reference for advancing proactive interventions in medical dispute management, with potential for adaptation alongside global best practices.

## 5 Conclusion

Findings from this research indicate a strong correlation between the introduction of PSM and enhanced CRR, coupled with a decline in IP at the two campuses of ZPHCM, evidenced by substantial effect magnitudes (Cohen’s d > 1.1). A pronounced growth in patient satisfaction was also observed. Through the synthesis of preemptive communication, institutional risk mitigation, and systematic quality assurance, PSM constitutes a localized framework for preempting medical disputes, tailored to operate within China’s administrative petition system and providing a supplementary option to established international models. Nevertheless, the present evidence, constrained by the single-center before-and-after design, limited sample size, and temporal data dependencies, cannot conclusively demonstrate causality. Subsequent investigations should seek to verify PSM’s independent efficacy utilizing designs such as multicenter stepped-wedge randomized trials and examine its transferability to other healthcare settings and institutional contexts.

## Data Availability

The original contributions presented in the study are included in the article/Supplementary material, further inquiries can be directed to the corresponding authors.
